# Voluntary running improves synaptic degeneration of the anterior cingulate cortex in knee osteoarthritis

**DOI:** 10.1186/s13041-025-01207-9

**Published:** 2025-08-22

**Authors:** Ryo Miyake, Manabu Yamanaka, Wataru Taniguchi, Naoko Nishio, Yuki Matsuyama, Takeru Ueno, Yuta Kaimochi, Terumasa Nakatsuka, Hiroshi Yamada

**Affiliations:** 1 Department of Orthopaedic Surgery, Wakayama Medical University, Wakayama, Japan; 2 Pain Research Center, Kansai University of Health Sciences, Osaka, Japan

**Keywords:** Osteoarthritis of the knee, Chronic pain, Synaptic plasticity, Anterior cingulate cortex, Exercise therapy, Voluntary running, Pain-escape, Pre-long-term potentiation, Post-long-term potentiation

## Abstract

Osteoarthritis of the knee (knee OA) causes chronic pain involving peripheral tissues, the spinal cord, and the brain. Neuropathic pain leads to changes in synaptic plasticity in the anterior cingulate cortex (ACC). However, whether such changes occur in knee OA mice and their association with exercise therapy remains unclear. Therefore, this study investigated these aspects using electrophysiological and behavioral approaches. We found no induction of pre- or post-long-term potentiation (LTP) in the ACC of knee OA mice. Application of ZD7288 and zeta inhibitory peptide (ZIP) reduced the amplitude of evoked excitatory postsynaptic currents, indicating pre-existing changes in synaptic plasticity in the ACC. Microinjection of ZD7288 and ZIP improved pain-escape and anxiety-like behaviors. Voluntary running exercise induced pre- and post-LTP and improved these behaviors in knee OA mice. Exercise therapy for knee OA may alter synaptic plasticity in the ACC, contributing to behavioral improvements.

**Supplementary Information:**

The online version contains supplementary material available at 10.1186/s13041-025-01207-9.

## Introduction

Knee osteoarthritis (knee OA) causes chronic pain [1]. Total knee arthroplasty (TKA) provides excellent pain relief and treatment results [2]; however, the annual number of TKA procedures is predicted to reach 3.48 million by 2030 (673% increase from 2005) [3], raising concerns regarding the burden on the medical economy [4]. Thus, symptom relief through conservative treatment is crucial. Despite the development of various analgesic drugs, no sufficient analgesic effects have been achieved in many patients; this is likely because chronic pain involves various factors [5]. Patients with chronic pain often suffer from emotional disorders, such as anxiety, which is believed to exacerbate chronic pain [6–8]. Serotonin/noradrenaline reuptake inhibitors are used to treat chronic pain and pain-related emotional disorders; however, there are concerns regarding the number of associated side effects [9–11]. Therefore, exploring alternative treatments with fewer side effects is important. Moderate exercise therapy has an excellent improvement effect on chronic pain and emotional disorders; thus, it is attracting attention [12–14].

The anterior cingulate cortex (ACC) has recently gained attention for its important role in chronic pain and emotional disorders [15–19]. Patients with knee OA and stronger anxiety have lower pain thresholds, and a behavioral experiment using rats demonstrated lower pain thresholds in anxious rats than in normal rats. Additionally, the ACC of rats with anxiety was activated based on immunohistological staining [20]. Furthermore, exercise therapy affects the ACC on functional magnetic resonance imaging (fMRI) [21]. It exerts pain-suppressing effects on knee OA via various brain regions, including the ACC [22, 23]. These findings indicate that ACC is associated with chronic pain and anxiety disorders caused by knee OA and that exercise therapy has some effects on ACC. We previously reported that ACC stimulation induces the enhancement and mechanism of synaptic transmission (long-term potentiation [LTP]: enhancement of synaptic plasticity in the central nervous system) [24–26]. Since the report of its association with memory and learning in the hippocampus [27–29], LTP has been studied in various brain regions, including the ACC and insular cortex [30]. Most studies on LTP have focused on post-LTP through activating postsynaptic glutamate receptors (N-methyl-D-aspartate receptors) [31]. In contrast, non-N-methyl-D-aspartate-mediated pre-LTP was recently reported in the ACC of the neuropathic model. Such LTP was suggested to play an important role in behavioral sensitization and anxiety disorders caused by chronic pain [24, 32]. However, there have been no studies on synaptic plasticity changes in the ACC of knee OA models or how synaptic plasticity in knee OA models is affected by exercise therapy. Therefore, we aimed to examine synaptic plasticity changes in the ACC of knee OA mice and their association with pain and anxiety behaviors using whole-cell patch-clamp recording, pharmacological, and behavioral techniques. In addition, we aimed to examine whether applying voluntary running exercise loads would improve synaptic plasticity changes in the ACC and improve the pain-escape and anxiety behaviors of knee OA mice.

## Results

### Knee osteoarthritis causes synaptic plasticity changes in the ACC

**Fig. 1:**
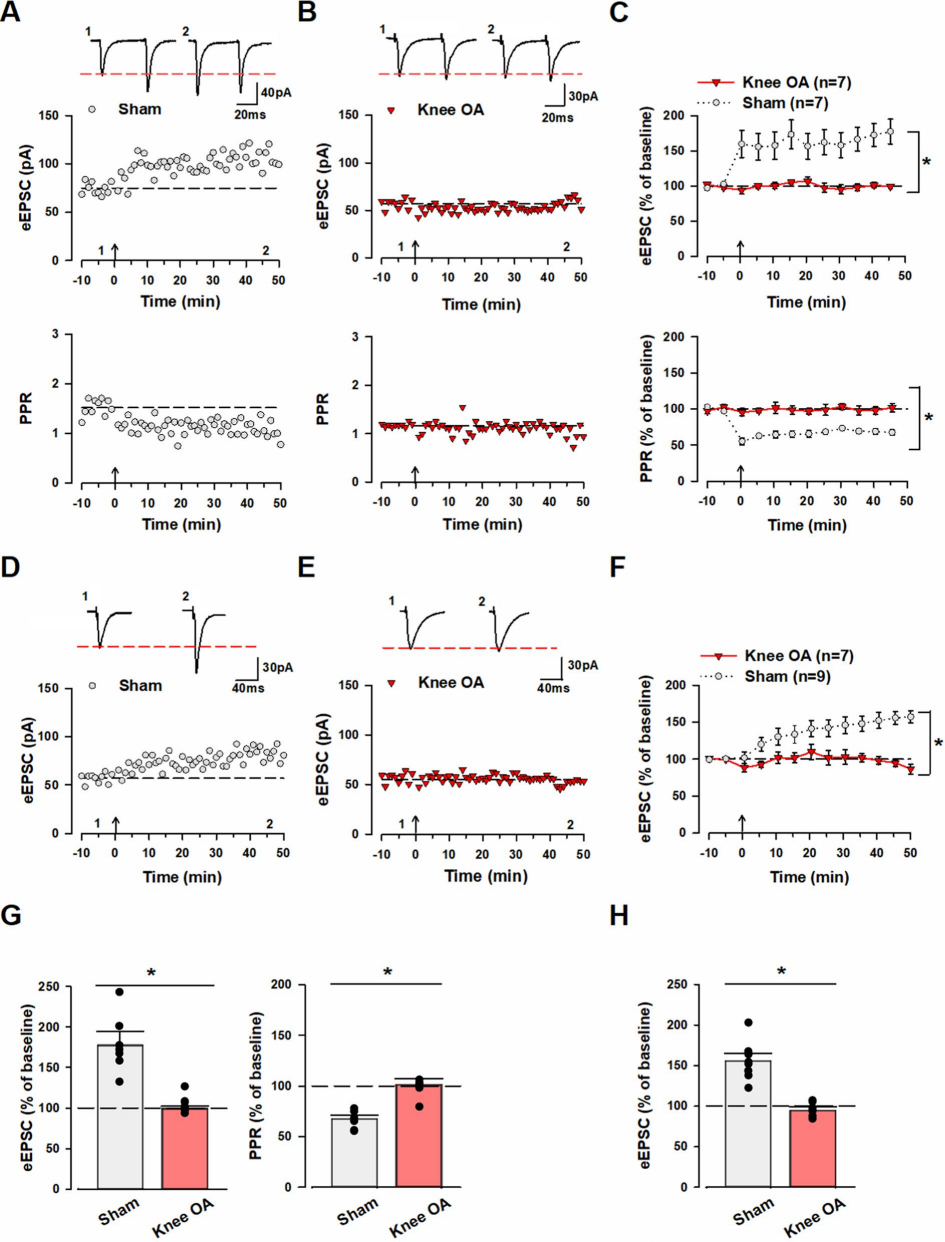
Induction of pre- and post-LTP is absent in knee OA mice. **A** Top: Sample traces of eEPSC in the ACC of sham model with paired-pulse stimulation at 50 ms interstimulus interval for 10 min during baseline (1) and 50 min after pairing protocol (2) at a holding membrane potential of − 60 mV. Middle: A time course plot of a representative single example. Bottom: A time course plot of the PPR for this neuron. The arrow donates the time of pre-LTP induction. **B** Top: Sample traces of eEPSC in the ACC of knee OA model with paired-pulse stimulation at 50 ms interstimulus interval for 10 min during baseline (1) and 50 min after pairing protocol (2) at a holding membrane potential of − 60 mV. Middle: A time course plot of a representative single example. Bottom: A time course plot of the PPR for this neuron. The arrow donates the time of pre-LTP induction. **C** Pooled data show that pre-LTP was induced in the sham group, whereas it was not induced in the knee OA group. Top: In the sham group (gray circle: n = 7 neurons/7 mice), pairing protocol stimulation increased the amplitude of eEPSC but resulted in no change in the knee OA group (red triangle: n = 7 neurons/7 mice). Bottom: In the sham group (gray circle: n = 7 neurons/7 mice), pairing protocol stimulation decreased PPR but resulted in no change in the knee OA group (red triangle: n = 7 neurons/7 mice). **D** Top: Sample traces of eEPSC in the ACC of sham model with single-pulse stimulation for 10 min during baseline (1) and 50 min after pairing protocol (2) at a holding membrane potential of − 60 mV. Bottom: A time course plot of a representative single example. **E** Top: Sample traces of eEPSC in the ACC of knee OA model with single-pulse stimulation for 10 min during baseline (1) and 50 min after pairing protocol (2) at a holding membrane potential of − 60 mV. Bottom: A time course plot of a representative single example. **F** Pooled data show that post-LTP was induced in the sham group, whereas it was not induced in the knee OA group. Top: In the sham group (gray circle: n = 9 neurons/9 mice), pairing protocol stimulation increased the amplitude of eEPSC, but it resulted in no change in the knee OA group (red triangle: n = 7 neurons/7 mice). **G** Summary of the amplitude of eEPSC and PPR after pre-LTP induction. The mean amplitudes of eEPSC and PPR were determined at 45–50 min after pre-LTP induction stimulation. **H** Summary of the amplitude of eEPSC after post-LTP induction. The mean amplitudes of eEPSC were determined at 45–50 min after post-LTP induction stimulation. Error bars represent SEM. * indicates a p-value of less than 0.05

The application of pairing protocol stimulation to pyramidal cell synapses in the ACC induces pre- and post-LTP [24–26, 33]. In the presence of the γ-aminobutyric acid type A (GABAA) receptor antagonist picrotoxin (100 µM), we stimulated layer V/VI of the ACC, and with the potential maintained at − 60 mV, we recorded evoked excitatory post-synaptic currents (eEPSC) in response to paired-pulse stimulation (pulse interval: 50 ms) or single-pulse stimulation in layer II/III pyramidal cells. Further, we recorded the paired-pulse ratio (PPR), which is commonly used as a presynaptic index. After recording a stable eEPSC baseline for 10 min, we applied the pairing protocol stimulation described above. The sham group showed an increase in the amplitude of eEPSC compared to baseline [45–50 min after pairing protocol stimulation: 177 ± 17.5% of baseline (Fig. 1A, C), 156 ± 8.7% of baseline (Fig. 1D, F)] and a decrease in PPR [45–50 min after pairing protocol stimulation: 67 ± 3.7% of baseline (Fig. 1A, C)]. These results indicate the induction of pre- and post-LTP, as seen in previous studies.

Furthermore, we examined the effects of knee OA on LTP in the ACC. Interestingly, the knee OA group showed no increase in the amplitude of eEPSC, even after each pairing protocol stimulation [45–50 min after pairing protocol stimulation: 99 ± 2.8% of baseline (Fig. 1B, C), 95 ± 4.6% of baseline (Fig. 1E, F)]. In addition, the knee OA group showed no decrease in PPR [45–50 min after pairing protocol stimulation: 102 ± 5.7% of baseline (Fig. 1B, C)]. A significant difference in the amplitude of eEPSC at 45–50 min after pairing protocol stimulation was observed between the knee OA and sham groups [knee OA group vs. sham group; amplitude of eEPSC: one-way analysis of variance (ANOVA), F (1.12) = 19.3, *p < 0.05 (Fig. 1C, G), F (1.14) = 33.0, *p < 0.05 (Fig. 1F, H)]. Moreover, the two groups showed a significant difference in PPR at 45–50 min after pairing protocol stimulation [PPR: one-way ANOVA, F (1.12) = 25.5, *p < 0.05, (Fig. 1C, G)]. These results suggest the loss of pre- and post-LTP in the ACC of knee OA mice.

**Fig. 2:**
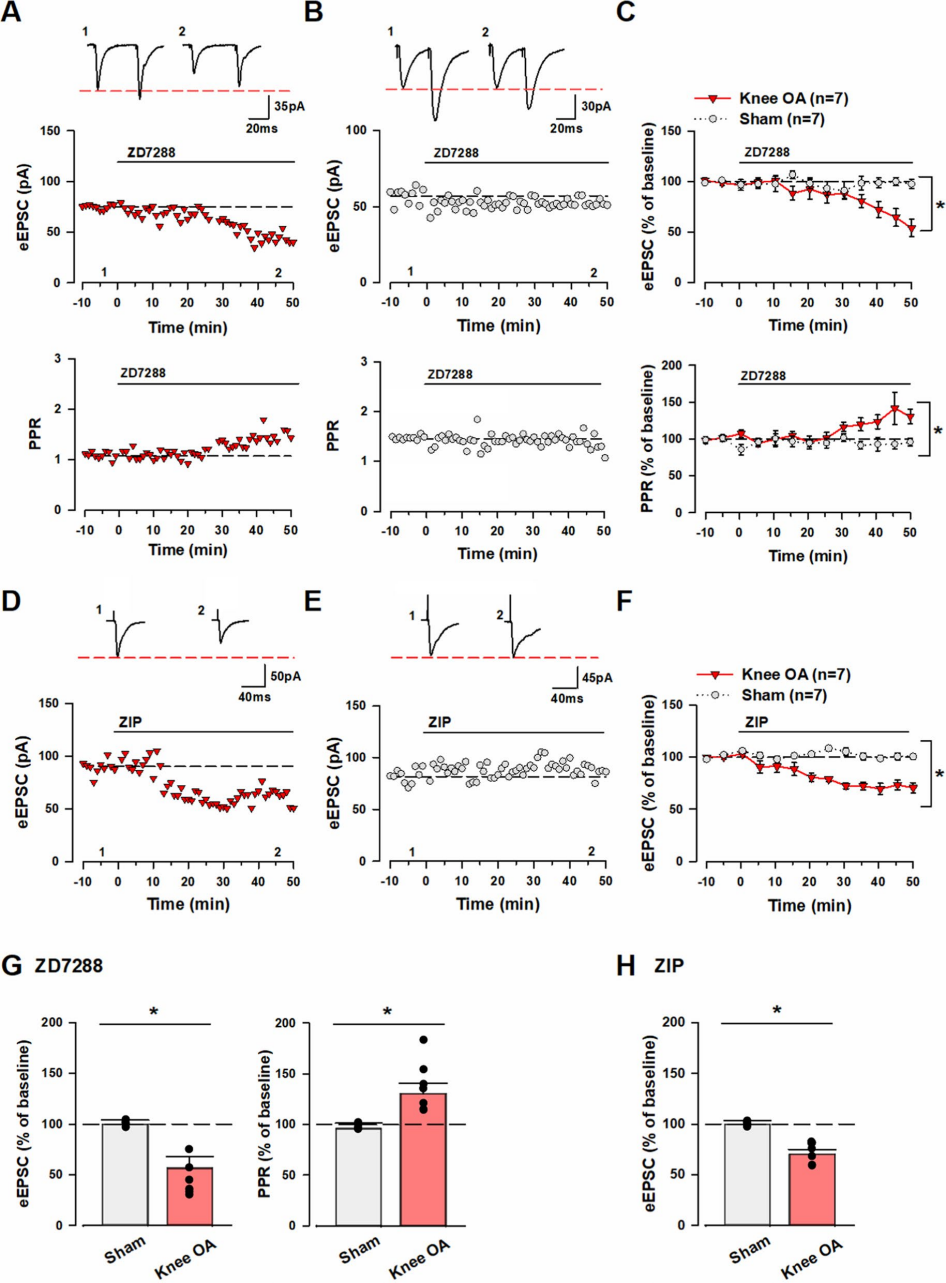
Knee OA induces pre- and post-LTP in the ACC. **A** Top: Sample traces of eEPSC in the ACC of knee OA model with paired-pulse stimulation at 50 ms interstimulus interval for 10 min during baseline (1) and 55 min after ZD7288 (10 µM) was applied (2) at a holding membrane potential of − 60 mV. Middle: A time course plot of a representative single example. Bottom: A time course plot of the PPR for this neuron. The arrow donates the time of pre-LTP induction. **B** Top: Sample traces of eEPSC in the ACC of sham model with paired-pulse stimulation at 50 ms interstimulus interval for 10 min during baseline (1) and 55 min after ZD7288 (10 µM) was administered (2) at a holding membrane potential of − 60 mV. Middle: A time course plot of a representative single example. Bottom: A time course plot of the PPR for this neuron. The arrow donates the time of pre-LTP induction. **C** Pooled data show that the ACC of knee OA mice had pre-existing pre-LTP. Top: The sham group (gray circle: n = 7 neurons/7 mice) showed no change in the amplitude of eEPSC after the perfusion administration of ZD7288 (10 µM). The knee OA group (red triangle: n = 7 neurons/7 mice) showed a decrease in the amplitude of eEPSC after the perfusion administration of ZD7288 (10 µM). Bottom: The sham group (gray circle: n = 7 neurons/7 mice) showed no change in PPR after the perfusion administration of ZD7288. However, the knee OA group (red triangle: n = 7 neurons/7 mice) showed an increase in PPR. **D** Top: Sample traces of eEPSC in the ACC of knee OA model with single-pulse stimulation for 10 min during baseline (1) and 55 min after ZIP (5 µM) was administered (2) at a holding membrane potential of − 60 mV. Middle: A time course plot of a representative single example. **E** Top: Sample traces of eEPSC in the ACC of sham model with single-pulse stimulation for 10 min during baseline (1) and 55 min after ZIP (5 µM) was administered (2) at a holding membrane potential of − 60 mV. Middle: A time course plot of a representative single example. **F** Pooled data show that the ACC of knee OA mice had pre-existing post-LTP. Top: The sham group (gray circle: n = 7 neurons/7 mice) showed no change in the amplitude of eEPSC after the perfusion administration of ZIP. However, the knee OA group (red triangle: n = 7 neurons/7 mice) showed a decrease in the amplitude of eEPSC after the perfusion administration of ZIP (5 µM). **G** Summary of the amplitude of eEPSC and PPR after ZD7288 (10 µM) was administered. The mean amplitudes of eEPSC and PPR were determined at 50–55 min after ZD7288 (10 µM) was administered. **H** Summary of the amplitude of eEPSC after ZIP (5 µM) was administered. The mean amplitudes of eEPSC were determined at 50–55 min after ZIP (5 µM) was administered. Error bars represent SEM. * indicates a p-value of less than 0.05

We considered two possible causes of the loss of pre- and post-LTP in the ACC of knee OA mice: inhibition and pre-existing induction of LTP. Hyperpolarization-activated cyclic nucleotide-gated (HCN) channels and protein kinase M zeta (PKMζ) are associated with the maintenance of pre- and post-LTP, respectively, in a neuropathic model [24, 34]. Thus, we examined the sensitivity of ACC neurons of knee OA mice to ZD7288, an HCN channel blocker, and zeta inhibitory peptide (ZIP), a PKMζ inhibitor. After recording a stable eEPSC baseline in response to paired-pulse (pulse interval: 50 ms) or single-pulse stimulation for 10 min, we administered the mice with ZD7288 (10 µM) or ZIP (5 µM) by perfusion and recorded eEPSC. Notably, the results showed that ZD7288 and ZIP decreased the amplitude of eEPSC in the ACC neurons in the knee OA group [50–55 min after perfusion administration of ZD7288: 54 ± 8.7% of baseline (Fig. 2A, C), 50–55 min after perfusion administration of ZIP: 71 ± 4.9% of baseline (Fig. 2D, F)]. However, neither ZD7288 nor ZIP affected the amplitude of eEPSC in the sham group [50–55 min after perfusion administration of ZD7288: 99 ± 1.0% of baseline (Fig. 2B, C), 50–55 min after perfusion administration of ZIP: 101 ± 3.1% of baseline (Fig. 2E, F)]. In addition, ZD7288 increased PPR in the knee OA group [50–55 min after perfusion administration of ZD7288: 138 ± 9.7% of baseline (Fig. 2A, C)]. In contrast, ZD7288 had no effect on PPR in the sham group [50–55 min after perfusion administration of ZD7288: 96 ± 1.9% of baseline (Fig. 2B, C)]. A significant difference in the amplitude of eEPSC at 50–55 min after perfusion administration of ZD7288 and ZIP was observed between the knee OA and sham groups [knee OA group vs. sham group; amplitude of eEPSC: one-way ANOVA, F (1.12) = 27.8, *p < 0.05 (Fig. 2C, G), F (1.12) = 27.1, *p < 0.05 (Fig. 2F, H)]. Furthermore, the two groups showed a significant difference in PPR at 50–55 min after perfusion administration of ZD728 [knee OA group vs. sham group; PPR: one-way ANOVA, F (1.12) = 17.9, *p < 0.05, (Fig. 2C, G)]. These results suggest that ACC neurons of knee OA mice had pre-existing plasticity changes that enhanced excitatory synaptic transmission.

### Microinjection of ZD7288 and ZIP into the ACC improves pain-escape and anxiety-like behaviors of knee OA mice

**Fig. 3:**
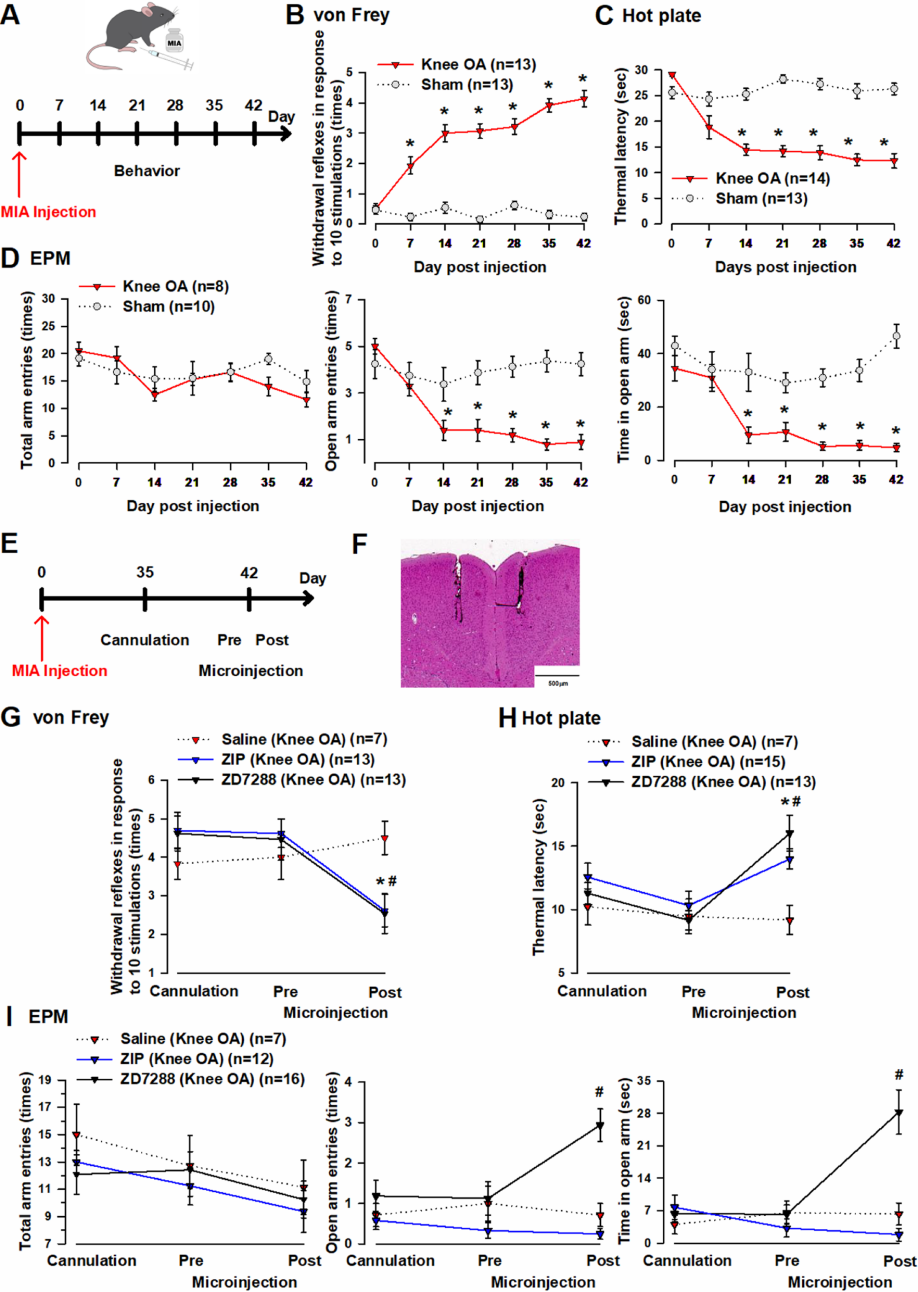
Knee OA mice exhibit pain-escape and anxiety-like behaviors, which are suppressed by microinjection of ZD7288 and ZIP into the ACC. **A** Behavioral evaluation timeline. **B** The knee OA group (red triangle: n = 13 mice) showed a significant increase in the number of withdrawal reflexes of the right hindlimb compared to the sham group (gray circle: n = 13 mice) from 1 week after MIA administration. **C** Compared with the sham group (gray circle: n = 13 mice), the knee OA group (red triangle: n = 14 mice) showed a significant difference in the time until the appearance of a noxious sign from 2 weeks after MIA administration. **D** Compared with the sham group (gray circle: n = 10 mice), the knee OA group (red triangle: n = 8 mice) showed no significant difference in the number of total arm entries but showed significant decreases in the number of open arm entries and the time staying in an open arm from 2 weeks after MIA administration. **E** Behavioral evaluation timeline, including cannulation and microinjection. **F** Bilateral microinjection sites for ZD7288 and ZIP in the ACC. **G** After microinjection, the knee OA (ZIP microinjection) group (blue triangle: n = 13 mice) showed a significant decrease in the number of withdrawal reflexes of the right hindlimb compared to the knee OA (saline microinjection) group (red triangle: n = 7 mice). In addition, after microinjection, the knee OA (ZD7288 microinjection) group (black triangle: n = 13 mice) showed a significant decrease in the number of withdrawal reflexes of the right hindlimb compared to the knee OA (saline microinjection) group (gray circle: n = 7 mice). **H** After microinjection, the knee OA (ZIP microinjection) group (blue triangle: n = 15 mice) showed a significant decrease in the time until the appearance of a noxious sign compared to the knee OA (saline microinjection) group (red triangle: n = 7 mice). Moreover, after microinjection, the knee OA (ZD7288 microinjection) group (black triangle: n = 13 mice) showed a significant decrease in the time until the appearance of a noxious sign compared to the knee OA (saline microinjection) group (gray circle: n = 7 mice). **I** After microinjection, the knee OA (ZD7288 microinjection) group showed no significant difference in the number of total arm entries compared to the knee OA (saline microinjection) group but showed significant increases in the number of open arm entries and the time staying in the arm. Error bars represent SEM. * and # indicate a p-value of less than 0.05

Knee OA models exhibit pain-escape and anxiety-like behaviors [35–38]. In the present study, we evaluated pain-escape and anxiety-like behaviors of knee OA mice using the von Frey, hot plate, and Elevated Plus Maze (EPM) tests (Fig. 3A). In the von Frey test, a significant difference in the number of withdrawal reflexes of the right hindlimb from 1 week after monoiodoacetic acid (MIA) administration was observed between the knee OA and sham groups (knee OA group: 1.5 ± 0.3 times, sham group: 0.2 ± 0.1 times, one-way ANOVA, F (1.24) = 19.7, *p < 0.05, Fig. 3A). A significant difference was observed up to 6 weeks after MIA administration (knee OA group: 4.2 ± 0.3 times, sham group: 0.2 ± 0.1 times, one-way ANOVA, F (1.24) = 170.8, *p < 0.05, Fig. 3A).

In the hot plate test, a significant difference in the time until the appearance of a noxious sign from 2 weeks after MIA administration was observed between the knee OA and sham groups (knee OA group: 14.4 ± 1.1 s, sham group: 25.3 ± 1.0 s, one-way ANOVA, F (1.25) = 53.1, *p < 0.05, Fig. 3C). A significant difference was observed up to 6 weeks after MIA administration (knee OA group: 12.3 ± 1.4 s, sham group: 26.3 ± 1.0 s, one-way ANOVA, F (1.25) = 63.8, *p < 0.05, Fig. 3B). In the EPM test, the knee OA and sham groups showed no significant difference in the number of total arm entries; however, significant differences were observed in the number of open arm entries (knee OA group: 1.4 ± 0.4 times, sham group: 3.4 ± 2.1 times, one-way ANOVA, F (1.16) = 6.0, *p < 0.05, Fig. 3D) and the time staying in the arm (knee OA group: 9.5 ± 3.1 s, sham group: 33.1 ± 7.1 s, one-way ANOVA, F (1.16) = 10.9, *p < 0.05, Fig. 3D) from 2 weeks following MIA administration. The significant differences in the number of open arm entries (knee OA group: 0.9 ± 0.3 times, sham group: 4.3 ± 0.5 times, one-way ANOVA, F (1.16) = 35.6, *p < 0.05, Fig. 3D) and the time staying in the arm (knee OA group: 4.8 ± 1.6 s, sham group: 46.6 ± 4.5 s, one-way ANOVA, F (1.16) = 92.7, *p < 0.05, Fig. 3D) were observed up to 6 weeks after MIA administration. These results show that knee OA mice exhibited pain-escape and anxiety-like behaviors, as seen in previous studies.

We electrophysiologically demonstrated that knee OA mice had pre-existing synaptic plasticity changes in the ACC. Thus, we tested the hypothesis that synaptic plasticity changes might be associated with pain-escape and anxiety-like behaviors. Using the von Frey, hot plate, and EPM tests, we examined changes in pain-escape and anxiety-like behaviors of knee OA mice before and after microinjection of ZD7288 and ZIP into the ACC 6 weeks after MIA administration (Fig. 3E). After the behavioral experiment, the brains were removed, sliced, and stained with hematoxylin and eosin (HE) to confirm the exact injection position (Fig. 3F). In the von Frey test, the knee OA (ZIP microinjection), knee OA (ZD7288 microinjection), and knee OA (saline microinjection) groups showed no significant difference in the number of withdrawal reflexes of the right hindlimb before microinjection (5 weeks after MIA administration). However, after microinjection, the knee OA (ZIP microinjection) and knee OA (ZD7288 microinjection) groups showed a significant difference in the number of withdrawal reflexes of the right hindlimb compared to the knee OA (saline microinjection) group (knee OA [ZIP microinjection] group: 2.5 ± 1.9 times, knee OA [ZD7288 microinjection] group: 2.6 ± 1.5 times, knee OA [saline microinjection] group: 4.7 ± 1.1 times, one-way ANOVA, F (2.30) = 5.0, *p < 0.05, Fig. 3H). In the hot plate test, the knee OA (ZIP microinjection), knee OA (ZD7288 microinjection), and knee OA (saline microinjection) groups showed no significant difference in the time until the appearance of a noxious sign before microinjection (5 weeks after MIA administration). Conversely, after microinjection, the knee OA (ZIP microinjection) and knee OA (ZD7288 microinjection) groups showed a significant difference in the time until the appearance of a noxious sign, compared to the knee OA (saline microinjection) group (knee OA [ZIP microinjection] group: 16.0 ± 1.4 s, knee OA [ZD7288 microinjection] group: 14.0 ± 0.8 s, knee OA [saline microinjection] group: 9.6 ± 1.1 s, one-way ANOVA, F (2.32) = 5.9, *p < 0.05, Fig. 3I). In the EPM test, the knee OA (ZIP microinjection), knee OA (ZD7288 microinjection), and knee OA (saline microinjection) groups showed no significant difference in the number of total arm entries, number of open arm entries, or time staying in the arm before microinjection (5 weeks after MIA administration). However, although there was no significant difference in the number of total arm entries after microinjection, the knee OA (ZD7288 microinjection) group showed significant differences in the number of open arm entries (knee OA [ZIP microinjection] group: 0.3 ± 0.1 times, knee OA [ZD7288 microinjection] group: 2.9 ± 0.4 times, knee OA [saline microinjection] group: 0.7 ± 0.3 times, one-way ANOVA, F (2.32) = 20.1, *p < 0.05, Fig. 3J) and the time staying in the arm (knee OA [ZIP microinjection] group: 1.8 ± 1.4 s, knee OA [ZD7288 microinjection] group: 28.3 ± 4.8 s, knee OA [saline microinjection] group: 6.3 ± 2.4 s, one-way ANOVA, F (2.32) = 14.5, *p < 0.05, Fig. 3J) compared to the knee OA (ZIP microinjection) and knee OA (saline microinjection) groups. As a result, the knee OA (ZD7288 microinjection) group showed significant improvement in pain-escape and anxiety-like behaviors, whereas the knee OA (ZIP microinjection) group only showed significant improvement in pain-escape behavior. However, the sham group microinjected with ZD7288, ZIP, or physiological saline showed no significant difference in pain-escape or anxiety-like behaviors (Additional file 1A, B, C). These results indicate that synaptic plasticity changes in the ACC of knee OA mice are associated with pain-escape and anxiety-like behaviors.

### Voluntary running exercise load improves pain-escape and anxiety-like behaviors in knee OA mice

**Fig. 4:**
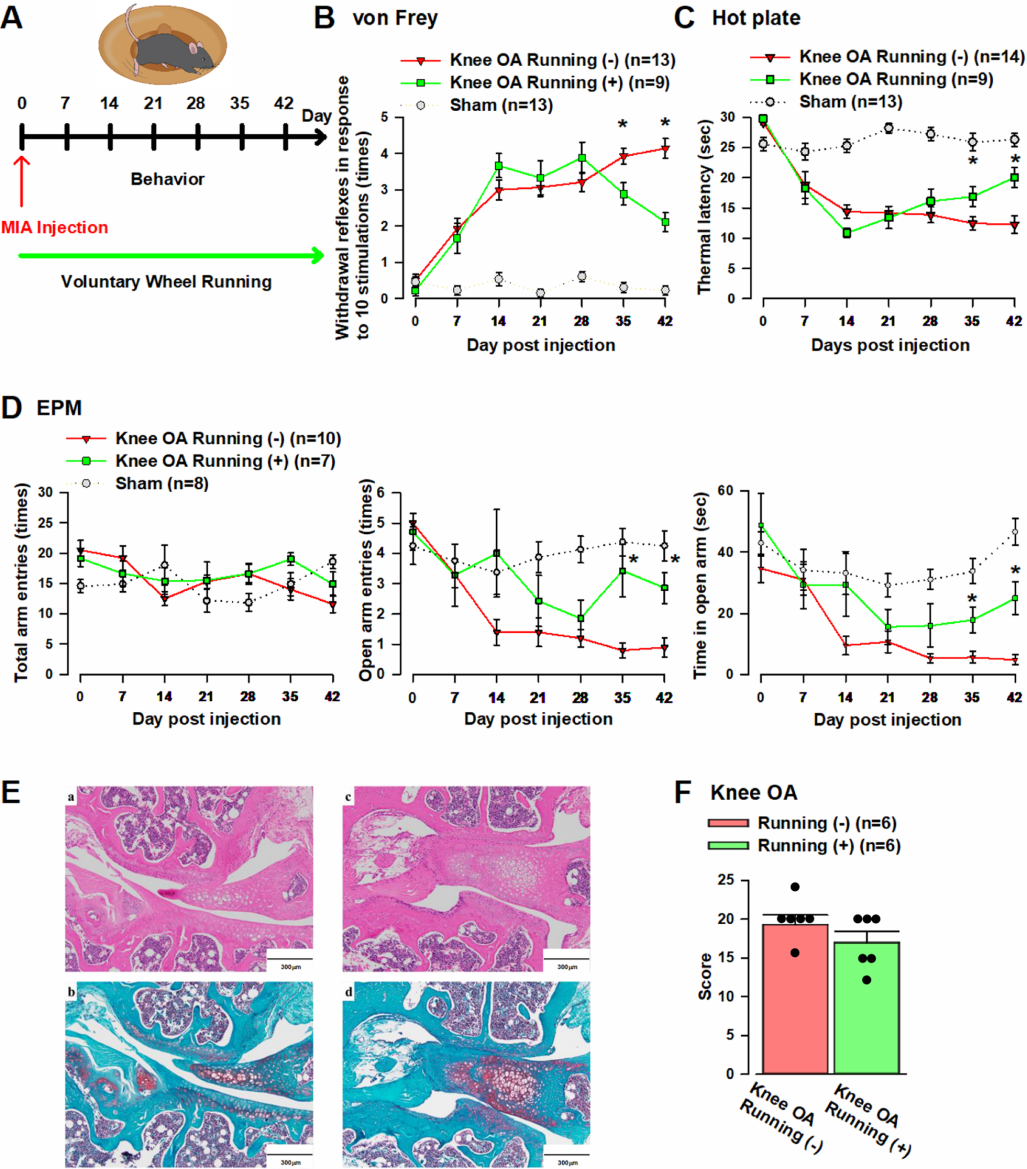
The application of voluntary running exercise improves pain-escape and anxiety-like behaviors in knee OA mice. **A** Duration of voluntary running exercise load and behavioral evaluation timeline. **B** Compared to the knee OA (– voluntary running exercise load) group, the knee OA (+ voluntary running exercise load) group showed a significant decrease in the number of withdrawal reflexes of the right hindlimb from the fifth week of voluntary running exercise load. **C** Compared with the knee OA (– voluntary running exercise load) group, the knee OA (+ voluntary running exercise load) group showed a significant increase in the time until the appearance of a noxious sign from the fifth week of voluntary running exercise load. **D** Compared with the knee OA (– voluntary running exercise load) group, the knee OA (+ voluntary running exercise load) group showed no significant difference in the number of total arm entries but showed significant increases in the number of open arm entries and the time staying in the arm from the fifth week of voluntary running exercise load. **E** a: HE staining image of the right knee joint of knee OA (– voluntary running exercise load) mice. b: Safranin O-fast green staining image of the right knee joint of knee OA (– voluntary running exercise load) mice. c: HE staining image of the right knee joint of knee OA (+ voluntary running exercise load) mice. d: Safranin O-fast green staining image of the right knee joint of knee OA (+ voluntary running exercise load) mice. **F** The knee OA (– voluntary running exercise load) group (n = 6) and the knee OA (+ voluntary running exercise load) group (n = 6) showed no clear significant difference in OARSI score. Error bars represent SEM. * indicates a p-value of less than 0.05

Clinically, exercise therapy is known to alleviate pain in knee OA patients and exert an antianxiety effect [39–41]. Furthermore, animal studies have reported that voluntary running exercise improves pain-escape and anxiety-like behaviors caused by knee OA [42, 43]. The pain-suppressing effect of exercise therapy is called exercise-induced hypoalgesia (EIH) [22]. With reference to previous studies, we conducted a behavioral experiment to examine the effect of voluntary running exercise load on pain-escape and anxiety-like behaviors of knee OA mice. After MIA administration, we placed the mice in a cage with a wireless running wheel (ENV-047; Med Associates) and subjected them to voluntary running exercise load for 6 weeks (Additional File 1D). Using the EPM, von Frey, and hot plate tests, we examined changes in their pain-escape and anxiety-like behaviors (Fig. 4A).

In the von Frey test, a significant difference was observed in the number of withdrawal reflexes of the right hindlimb between the knee OA (+ voluntary running exercise load) and knee OA (– voluntary running exercise load) groups (knee OA [– voluntary running exercise load] group: 3.9 ± 0.2 times, knee OA [+ voluntary running exercise load] group: 2.9 ± 0.3 times, one-way ANOVA, F (1.20) = 7.2, *p < 0.05, Fig. 4D) from the fifth week of voluntary running exercise load. A significant difference was observed up to the sixth week of voluntary running exercise load (knee OA [– voluntary running exercise load] group: 4.2 ± 0.3 times, knee OA [+ voluntary running exercise load] group: 2.1 ± 0.3 times, one-way ANOVA, F (1.20) = 27.8, *p < 0.05, Fig. 4B).

In the hot plate test, a significant difference was observed in the time until the appearance of a noxious sign between the knee OA (+ voluntary running exercise load) and knee OA (– voluntary running exercise load) groups (knee OA [– voluntary running exercise load] group: 12.5 ± 1.1 s, knee OA [+ voluntary running exercise load] group: 16.9 ± 1.6 s, one-way ANOVA, F (1.21) = 5.4, *p < 0.05, Fig. 4C) from the fifth week of voluntary running exercise load. The significant difference observed was up to the sixth week of voluntary running exercise load (knee OA [– voluntary running exercise load] group: 12.3 ± 1.4 s, knee OA [+ voluntary running exercise load] group: 20.1 ± 1.7 s, one-way ANOVA, F (1.21) = 12.2, *p < 0.05, Fig. 4D).

In the EPM test, the knee OA (+ voluntary running exercise load) and knee OA (– voluntary running exercise load) groups showed no significant difference in the number of total arm entries. Conversely, there were significant differences in the number of open arm entries (knee OA [– voluntary running exercise load] group: 0.8 ± 0.2 times, knee OA [+ voluntary running exercise load] group: 3.4 ± 0.9 times, one-way ANOVA, F (1.15) = 11.4, *p < 0.05, Fig. 4F) and the time staying in the arm (knee OA [– voluntary running exercise load] group: 5.6 ± 1.9 s, knee OA [+ voluntary running exercise load] group: 17.9 ± 4.2 s, one-way ANOVA, F (1.15) = 8.8, *p < 0.05, Fig. 4D) from the fifth week of voluntary running exercise load. The significant differences in the number of open arm entries (knee OA [– voluntary running exercise load] group: 0.9 ± 0.3 times, knee OA [+ voluntary running exercise load] group: 2.9 ± 0.5 times, one-way ANOVA, F (1.15) = 12.0, *p < 0.05, Fig. 4D) and the time staying in the arm (knee OA [– voluntary running exercise load] group: 4.8 ± 1.6 s, knee OA [+ voluntary running exercise load] group: 24.9 ± 5.3 s, one-way ANOVA, F (1.15) = 17.4, *p < 0.05, Fig. 4D) were observed up to the sixth week of voluntary running exercise load. Notably, the results revealed that the knee OA (+ voluntary running exercise load) group experienced improvements in pain-escape and anxiety-like behaviors from the fifth week of voluntary running exercise load. After completing the behavioral study, the right knee joints of the knee OA (– voluntary running exercise load) and knee OA (+ voluntary running exercise load) groups were removed and stained with safranin O-fast green and HE (Fig. 4E). The knee OA (– voluntary running exercise load) [n = 6] and (+ voluntary running exercise load) groups [n = 6] showed no obvious significant difference in the Osteoarthritis Research Society International (OARSI) score (Fig. 4F), respectively.

### Voluntary running exercise load improves synaptic plasticity changes in ACC neurons caused by knee OA

**Fig. 5:**
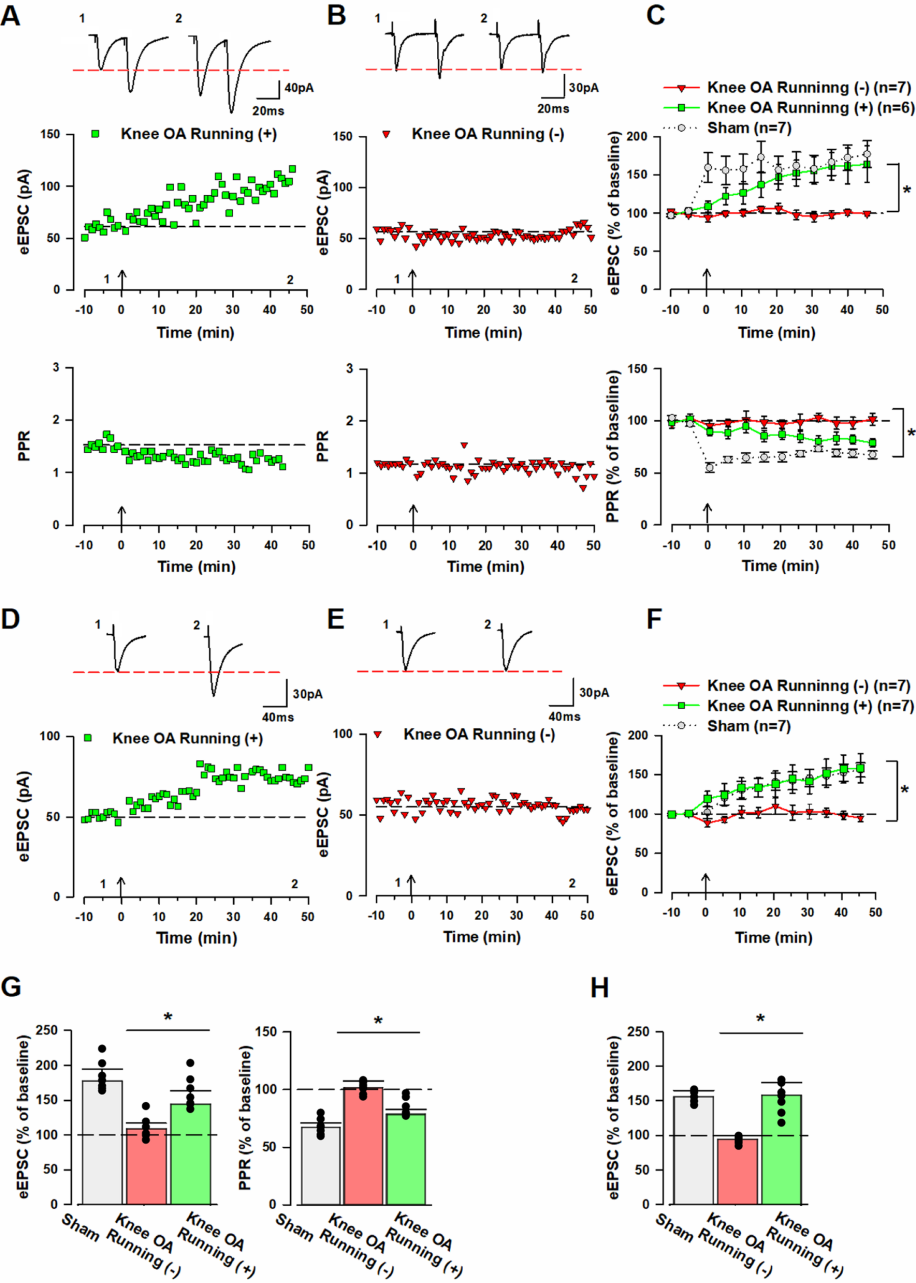
The application of voluntary running exercise for 6 weeks induces previously lost pre- and post-LTP in the ACC neurons of knee OA mice. **A** Top: Sample traces of eEPSC in the ACC of knee OA model (+ voluntary running exercise load) with paired-pulse stimulation at 50 ms interstimulus interval for 10 min during baseline (1) and 50 min after pairing protocol (2) at a holding membrane potential of − 60 mV. Middle: A time course plot of a representative single example. Bottom: A time course plot of the PPR for this neuron. The arrow donates the time of pre-LTP induction. **B** Top: Sample traces of eEPSC in the ACC of knee OA model (– voluntary running exercise load) with paired-pulse stimulation at 50 ms interstimulus interval for 10 min during baseline (1) and 50 min after pairing protocol (2) at a holding membrane potential of − 60 mV. Middle: A time course plot of a representative single example. Bottom: A time course plot of the PPR for this neuron. The arrow donates the time of pre-LTP induction. **C** Pooled data show that pre-LTP was not induced in the knee OA (– voluntary running exercise load) group, whereas it was induced in the knee OA (+ voluntary running exercise load) group. Top: In the knee OA (– voluntary running exercise load) group (red triangle: n = 7 neurons/7 mice), pairing protocol stimulation resulted in no change in the amplitude of eEPSC but resulted in an increase in the knee OA (+ voluntary running exercise load) group (green square: n = 6 neurons/6 mice). Bottom: In the knee OA group (red triangle: n = 7 neurons/7 mice), pairing protocol stimulation resulted in no change in PPR but resulted in a decrease in the knee OA (+ voluntary running exercise load) group (green square: n = 6 neurons/6 mice). **D** Top: Sample traces of eEPSC in the ACC of knee OA model (+ voluntary running exercise load) with single-pulse stimulation for 10 min during baseline (1) and 50 min after pairing protocol (2) at a holding membrane potential of − 60 mV. Bottom: A time course plot of a representative single example. **E** Top: Sample traces of eEPSC in the ACC of knee OA model (– voluntary running exercise load) with single-pulse stimulation for 10 min during baseline (1) and 50 min after pairing protocol (2) at a holding membrane potential of − 60 mV. Bottom: A time course plot of a representative single example. **F** Pooled data show that post-LTP was not induced in the knee OA (– voluntary running exercise load) group, whereas it was induced in the knee OA (+ voluntary running exercise load) group. Top: In the knee OA (– voluntary running exercise load) group (red triangle: n = 7 neurons/7 mice), pairing protocol stimulation resulted in no change in the amplitude of eEPSC but resulted in an increase in the knee OA (+ voluntary running exercise load) group (green square: n = 7 neurons/7 mice). **G** Summary of the amplitude of eEPSC and PPR after pre-LTP induction. The mean amplitudes of eEPSC and PPR were determined at 45–50 min after pre-LTP induction stimulation. **H** Summary of the amplitude of eEPSC and PPR after post-LTP induction. The mean amplitudes of eEPSC were determined at 45–50 min after the post-LTP induction stimulation. Error bars represent SEM. * indicates a p-value of less than 0.05

As described above, the application of voluntary running exercise led to improvements in pain-escape and anxiety-like behaviors of knee OA mice from the fifth week of exercise load. Based on this finding, we hypothesized that voluntary running exercise load would improve pre-existing synaptic plasticity changes in the ACC of knee OA mice; thus, we conducted an electrophysiological experiment to test the hypothesis. As mentioned above, the knee OA (– voluntary running exercise load) group showed no increase in the amplitude of eEPSC after each pairing protocol stimulation [45–50 min after pairing protocol stimulation: 99 ± 2.8% of baseline (Fig. 5B, C), 95 ± 4.6% of baseline (Fig. 5E, F)]. Additionally, the group showed no increase in PPR [45–50 min after pairing protocol stimulation: 102 ± 5.7% of baseline (Fig. 5B, C)]. However, the knee OA (+ voluntary running exercise load) group showed an increase in the amplitude of eEPSC following each pairing protocol stimulation [45–50 min after pairing protocol stimulation: 164 ± 24.2% of baseline (Fig. 5A, C), 45–50 min after pairing protocol stimulation: 158 ± 18.4% of baseline (Fig. 5D, F)]. In addition, the group showed a decrease in PPR [45–50 min after pairing protocol stimulation: 79 ± 4.1% of baseline (Fig. 5A, C)]. A significant difference in the amplitude of eEPSC at 45–50 min of pairing protocol stimulation was observed between the knee OA (+ voluntary running exercise load) and knee OA (– voluntary running exercise load) groups (knee OA [+ voluntary running exercise load] group vs. knee OA [– voluntary running exercise load] group; amplitude of eEPSC: one-way ANOVA, F (1.11) = 8.4, *p < 0.05, (Fig. 5C, G), F (1.12) = 11.1, *p < 0.05, (Fig. 5F, H)]. Additionally, the two groups showed a significant difference in PPR at 45–50 min after pairing protocol stimulation (knee OA [+ voluntary running exercise load] group vs. knee OA [– voluntary running exercise load] group; PPR: one-way ANOVA, F (1.11) = 9.9, *p < 0.05, [Fig. 5C, G]). These results demonstrated the induction of pre- and post-LTP, which was lost in the ACC neurons of knee OA mice, suggesting that voluntary running exercise load improved pre-existing synaptic plasticity changes in the ACC of knee OA mice.

## Discussion

In the present study, we found that pre- and post-LTP were lost in the ACC neurons of knee OA mice. Furthermore, the amplitude of eEPSC was decreased by administering an inhibitor (ZD7288) and a blocker (ZIP) of HCN channels and PKMζ, respectively, which are associated with the maintenance of LTP. This finding suggests the presence of pre-existing pre- and post-LTP, i.e., pre-existing synaptic plasticity changes in the ACC neurons of knee OA mice. Furthermore, our behavioral evaluation with the microinjection of ZD7288 and ZIP suggests that synaptic plasticity changes in the ACC may be involved in pain-escape and anxiety-like behaviors of knee OA mice. These findings revealed that the application of voluntary running exercise improved pain-escape and anxiety-like behaviors of knee OA mice and induced pre- and post-LTP, lost in the ACC neurons of knee OA mice, suggesting an improvement in synaptic plasticity changes.

### Loss of LTP in the ACC of knee OA mice

In recent years, two major types of LTP in the ACC have been reported: pre- and post-LTP. Additionally, in a neuropathic pain model, HCN channels and PKMζ have been reported to specifically maintain pre- and post-LTP, respectively. The present study showed that pre- and post-LTP were lost in the ACC of knee OA mice. Previous studies showed that pre- and post-LTP were lost in the ACC of a neuropathic model, and the analysis results of sensitivity to ZD7288 and ZIP indicated that pre- and post-LTP loss was caused by pre-existing synaptic plasticity changes [24, 26, 34]. Based on this, we hypothesized that HCN channels and PKMζ are also involved in the maintenance of pre-LTP and post-LTP in a knee OA model and conducted experiments to investigate this. In the present study, the administration of ZD7288 to ACC neurons of knee OA mice resulted in the loss of pre-LTP maintenance, a decrease in the amplitude of eEPSC, and an increase in PPR. Furthermore, the administration of ZIP resulted in the loss of post-LTP maintenance and a decrease in the amplitude of eEPSC. These results demonstrate that knee OA mice had pre-existing pre- and post-LTP, that is, pre-existing synaptic plasticity changes, in ACC neurons.

### Effects of microinjection of ZD7288 and ZIP into the ACC on pain-escape and anxiety-like behaviors in knee OA mice

ACC plays an important role in pain sensation and emotion. In particular, it is believed that presynaptic plasticity changes in the ACC of the neuropathic model are involved in lowering pain thresholds and emotional disorders; additionally, post-synaptic plasticity changes lower pain thresholds [19, 24, 44]. Our electrophysiological experiment showed pre-existing synaptic plasticity changes at ACC synapses of knee OA mice. Thus, to examine whether the synaptic plasticity changes were associated with pain-escape and anxiety-like behaviors, we microinjected ZD7288 and ZIP into the ACC of knee OA mice and conducted behavioral evaluation. A study using the neuropathic model reported that microinjection of ZD7288 into the ACC improved pain-escape and anxiety-like behaviors and that microinjection of ZIP improved only pain-escape behavior [24]. Similarly, our results showed that microinjection of ZD7288 into the ACC of knee OA mice improved pain-escape and anxiety-like behaviors, whereas microinjection of ZIP improved only pain-escape behavior. These results suggest that pre-existing synaptic plasticity changes in the ACC of knee OA mice were modulated by ZD7288 and ZIP, leading to improvements in pain-escape and anxiety-like behaviors.

### Effects of a voluntary running exercise load on pain-escape and anxiety-like behaviors in knee OA mice

Many studies have reported that exercise therapy improves pain and emotional disorders in the routine medical care of patients with knee OA [45, 46]. Because excessive exercise worsens knee OA, moderate exercise is recommended [47]. Additionally, animal studies have reported that voluntary running exercises on a running wheel and moderate treadmill training improve pain and anxiety [12, 48, 49]. In the present study, we subjected knee OA mice to a voluntary running exercise load using a running wheel (moderate exercise) and examined changes in their pain-escape and anxiety-like behaviors. The results showed that the application of a voluntary running exercise load for at least 5 weeks improved pain-escape and anxiety-like behaviors in knee OA mice. However, the knee OA (+ voluntary running exercise load) and knee OA (– voluntary running exercise load) groups showed no difference in the OARSI score, an index of histological degeneration of articular cartilage in the knee joint. In other words, the application of a voluntary running exercise load resulted in no change in the degree of knee OA, suggesting that pain-escape and anxiety-like behaviors were improved through a mechanism that affects central tissues.

### Effects of a voluntary running exercise load on LTP in knee OA mice

In recent years, many studies have reported that exercise therapy affects the brain and improves pain and emotional disorders [50, 51]. The association of this pain-suppressing effect of exercise therapy, known as EIH, with various brain regions has been suggested; however, there have been few studies on the relationship between exercise therapy and LTP. Although it was a field potential recording, a previous study reported that LTP was lost in the hippocampal dentate gyrus of sleep-deprived mice and that the application of a moderate exercise load induced LTP [52]. Another field potential recording study reported that LTP was lost in the ACC of the nociceptive model wherein complete Freund’s adjuvant (CFA) was administered to the hindlimbs of mice and that LTP was induced by a moderate exercise load [49]. Additionally, a study using MRI reported that exercise therapy activated the ACC of the cerebral cortex [21]. Therefore, we examined whether pre- and post-LTP, which were lost in the ACC of knee OA mice, would be induced after applying a voluntary running exercise load. Notably, pre- and post-LTP, which were lost in the ACC of knee OA mice, were induced after 6 weeks of voluntary running exercise. However, the involvement of serotonin has been reported in recent years. The ACC receives innervation from serotonergic terminals [53, 54], and serotonin suppresses excitatory synaptic transmission pre- and post-synaptically [55]. Furthermore, exercise therapy promoted serotonin secretion in the nociceptive model wherein CFA was administered to the hindlimbs of mice [49]. Exercise therapy may promote serotonin secretion at ACC synapses and suppress excitatory synapses; however, further studies on other possible mechanisms are needed.

## Limitation

There are some limitations to this study. First, we did not elucidate the mechanism by which exercise therapy induced LTP, which was lost in the ACC neurons of knee OA mice. Second, we were unable to investigate the effects of knee OA on h currents. Third, we did not determine whether knee OA alters the input–output relationship. Finally, it remains unclear how many ACC neurons underwent plastic changes and to what extent exercise therapy restored these plastic changes.

## Methods

All animal experiments, including animal care, were conducted after obtaining prior approval from the Animal Experiment Ethics Committee of Wakayama Medical University.

### Animals

Six-week-old (20–30 g) C57BL/6 male mice were purchased from Kiwa Laboratory Animals Co., Ltd. All mice were housed in groups of three to four and had free access to water and food under a 12-h light/dark cycle.

### Knee OA model

The knee OA mouse model was created as previously described [35]. Briefly, the mice were anesthetized with isoflurane, and the right knee joint was flexed and held with fingers. Thereafter, a needle was inserted under the patella perpendicular to the tibia, and an MIA solution dissolved in 0.9% sterile physiological saline (1 mg/10 µL) was administered into the right knee joint. Sham mice were administered 0.9% sterile physiological saline only. Mice in the electrophysiological experiment were used 6 weeks after MIA administration, and mice in the behavioral experiment were used 1–6 weeks after administration.

### Preparation of slices

Twelve-week-old mice were anesthetized with isoflurane, and coronal brain slices (300 µm) containing the ACC were collected using the conventional method [25, 26, 34, 56, 57]. These brain slices were transferred to a room-temperature chamber filled with artificial cerebrospinal fluid (ACSF) containing 124 mM NaCl, 2.5 mM KCl, 2 mM CaCl2, 1 mM MgSO4, 25 mM NaHCO3, 1 mM NaH2PO4, and 10 mM glucose; afterward, the brain slices were oxygenated (95% O2 and 5% CO2) for at least 1 h.

### Whole-cell patch-clamp recording

The experiment was performed in a recording chamber placed on the stage of a microscope (BX51WI OLYMPUS) equipped with an infrared differential interference contrast optical device for cell visualization. The potential was held at − 60 mV using Axopatch 200B Amplifier (Molecular Devices, CA). Layer V/VI of the ACC slices was stimulated every 30 s with a bipolar tungsten stimulation electrode, and eEPSC was recorded from layer II/III pyramidal neurons of the ACC. All electrophysiological procedures were performed in the presence of picrotoxin (100 µM), a GABAA receptor antagonist. A recording pipette (3–5 MΩ) was filled with a solution containing 145 mM K-gluconic acid, 5 mM NaCl, 1 mM MgCl2, 0.2 mM EGTA, 10 mM HEPES, 2 mM Mg-ATP, and 0.1 mM Na3-GTP (adjusted to a pH of 7.2 using KOH). Data were excluded if the access resistance maintained in the range of 15–30 MΩ changed by ≥ 15% during the experiment. The obtained data were filtered at 1 kHz and digitized at 10 kHz. Pre-LTP was induced using a previously reported pairing protocol [24, 26]. The pre-LTP induction protocol applies 2-Hz pulse stimulation 240 times while holding the postsynaptic cells at − 60 mV. Additionally, post-LTP was induced using a previously reported pairing protocol [25, 33, 58]. The post-LTP induction protocol applies 2-Hz pulse stimulation 80 times while holding the postsynaptic cells at + 30 mV. The eEPSC amplitude was adjusted to a range of 50–100 pA, and eEPSC was recorded to obtain a 10-min baseline before LTP induction. LTP was induced within 12 min of whole-cell patch clamp to avoid washing out intracellular components necessary for establishing synaptic plasticity.

### Cannulation and microinjection

Cannulation and microinjection of the mice were performed using the conventional method [24, 33, 34]. After the mice were anesthetized with isoflurane, each head was fixed with a stereotaxic apparatus. An incision was made in the head to expose the skull surface, and two small openings were made above the ACC. While carefully avoiding the dura mater, a 24-gauge guide cannula was inserted into the bilateral ACC (0.7 mm anterior to the bregma, ± 0.4 mm lateral to the midline, and 1.7 mm below the skull surface). A 30-gauge injection cannula was placed 0.1 mm below the guide cannula. After cannula placement, the mice were given a minimum of 1 week for recovery.

Microinjection was performed using an electric microinjector (IMS-30; Narishige) and a Hamilton syringe (Hamilton, Reno, NV, USA). First, 0.5 µL of ZD7288 (0.1 µg) or ZIP (10 nmol/µL) dissolved in 0.9% sterile physiological saline or sterile physiological saline alone was microinjected into each side of the ACC of the cannulated mice over 1 min. The injector remained in place for an additional minute to prevent backflow along the guide. After microinjection, the mice were returned to their familiar environment for 15 min, after which the behavioral experiment was performed. After the behavioral experiment, the brains were removed, sliced, and stained with HE to confirm the exact injection position (Fig. 3F).

### Behavioral experiment on pain-escape and anxiety-like behaviors

Experimenters were blinded to the animals and drugs.

#### The EPM test

The EPM test was performed using the conventional method [59]. The EPM (Med Associates) consisted of four orthogonal arms with a platform where the arms intersected. While two open arms had no sidewalls, two closed arms had gray opaque sidewalls. The mice were allowed 30 min to acclimatize to the indoor environment, after which the EPM test was performed. The head, facing a closed arm, was placed on the platform part of the plus maze, and the number of entries and the time staying in each of the open and closed arms were recorded for 5 min.

#### The von Frey test

For the von Frey test, we used a previously reported method as a reference [35, 60]. The mice were placed in a 6 × 6 × 6 cm chamber on a wire mesh floor 30 min before the test. After confirming the disappearance of spontaneous movements, an identical stimulation (stimulated at intervals of at least 5 min to avoid hyperalgesia due to repeated stimulation) with 0.16 g von Frey filament was applied to the bottom of the right foot 10 times, and the frequency of withdrawal reflexes was measured.

#### The hot plate test

The hot plate test was performed using the conventional method [24]. Briefly, the mice were individually placed on a hot plate (IITC Life Science) controlled at a temperature of 55 ± 1 ℃. The time to the first noxious sign, licking of the front paw, or response of jumping to avoid heat was recorded, and the animals were immediately removed from the hot plate. The cutoff time was set at 30 s to avoid injury to the paw pad.

### Voluntary wheel running

After MIA administration, knee OA mice were housed in a cage equipped with a thin-type wireless running wheel (ENV-047; Med Associates) for 6 weeks. Wheel revolutions were monitored via a wireless USB interface hub (DIG-807; Med Associates) and recorded hourly in Wheel Manager software (SOF-860; Med Associates).

### Histopathology scoring

Six weeks after MIA administration, knee OA mice (with or without voluntary running exercise load) were anesthetized with 5% isoflurane and, thereafter, sacrificed by decapitation. The harvested right knee joints were immersed and fixed in 10% formalin solution and decalcified with 10% ethylenediaminetetraacetic acid. The knee joints were embedded in paraffin, and two tissue sections were prepared. For histological evaluation of the cartilage, sections were stained with HE and safranin O-fast green and scored using the OARSI histopathology grading system. At least two blinded individuals independently scored the stained sections of each joint to determine a consensus score.

### Pharmacological inhibition

MIA, picrotoxin, ZD7288, and ZIP were purchased from Sigma-Aldrich. MIA was dissolved in 0.9% sterile physiological saline. For the electrophysiological experiment, picrotoxin and ZD7288 were dissolved in dimethyl sulfoxide (DMSO), and ZIP was dissolved in distilled water. For the behavioral experiment, ZD7288 and ZIP were dissolved in 0.9% sterile physiological saline.

### Data analysis

Data collection and analysis were performed using the Clampex 10.2 and Clampfit 10.2 software suite (Molecular Devices). ANOVA was used to compare two or more groups. Significance between groups was tested using Tukey’s test to adjust for multiple comparisons. All data were expressed as means ± standard error of the mean (SEM). In all cases, *p* < 0.05 was considered statistically significant.

## Supplementary Information

Additional file 1. Sham mice did not exhibit pain-escape or anxiety-like behaviors, which did not change after microinjection of ZD7288 and ZIP into the ACC. (A) After microinjection, the sham (ZIP microinjection) group (black circle: n = 9 mice) showed no significant difference in the number of withdrawal reflexes of the right hindlimb compared to the sham (saline microinjection) group (gray circle: n = 7 mice). In addition, after microinjection, the sham (ZD7288 microinjection) group (blue circle: n = 10 mice) showed no significant difference in the number of withdrawal reflexes of the right hindlimb compared to the sham (saline microinjection) group (gray circle: n = 7 mice). (B) After microinjection, the sham (ZIP microinjection) group (black circle: n = 9 mice) showed no significant difference in the time until the appearance of a noxious sign compared to the sham (saline microinjection) group (gray circle: n = 7 mice). Moreover, after microinjection, the sham (ZD7288 microinjection) group (blue circle: n = 10 mice) showed no significant difference in the time until the appearance of a noxious sign compared to the sham (saline microinjection) group (gray circle: n = 7 mice). (C) After microinjection, the sham (ZD7288 microinjection) group (blue circle: n = 8 mice) showed no significant difference in the number of total arm entries, open arm entries, and the time staying in the arm compared to the sham (saline microinjection) group (gray circle: n = 7 mice). (D) The number of running wheel revolutions in the knee OA (+ voluntary running exercise load) group (green square: n = 4 mice) was 20.3 (10^3^) ± 4.5 (10^3^) revolutions from 5–6 weeks after voluntary running exercise load.

## Data Availability

No datasets were generated or analysed during the current study.

## Abbreviations

ACC: Anterior cingulate cortex
ACSF: Artificial cerebrospinal fluid
ANOVA: One-way analysis of variance
eEPSC: Evoked excitatory postsynaptic current
EIH: Exercise-induced hypoalgesia
EPM: Elevated Plus Maze
fMRI: Functional magnetic resonance imaging
GABAA: γ-Aminobutyric acid type A
HCN: Hyperpolarization-activated cyclic nucleotide-gated
HE: Hematoxylin and eosin
Knee OA: Osteoarthritis of the knee
LTP: Long-term potentiation
MIA: Monoiodoacetic acid
OARSI: Osteoarthritis Research Society International
PKMζ: Protein kinase M zeta
PPR: Paired-pulse ratio
TKA: Total knee arthroplasty
ZIP: Zeta inhibitory peptide
