# Supplementary figures and images for: Voluntary running improves synaptic degeneration of the anterior cingulate cortex in knee osteoarthritis
Source: Mol Brain. 2025 Aug 22;18:72. doi: 10.1186/s13041-025-01207-9 (PMC12374455; doi:10.1186/s13041-025-01207-9)

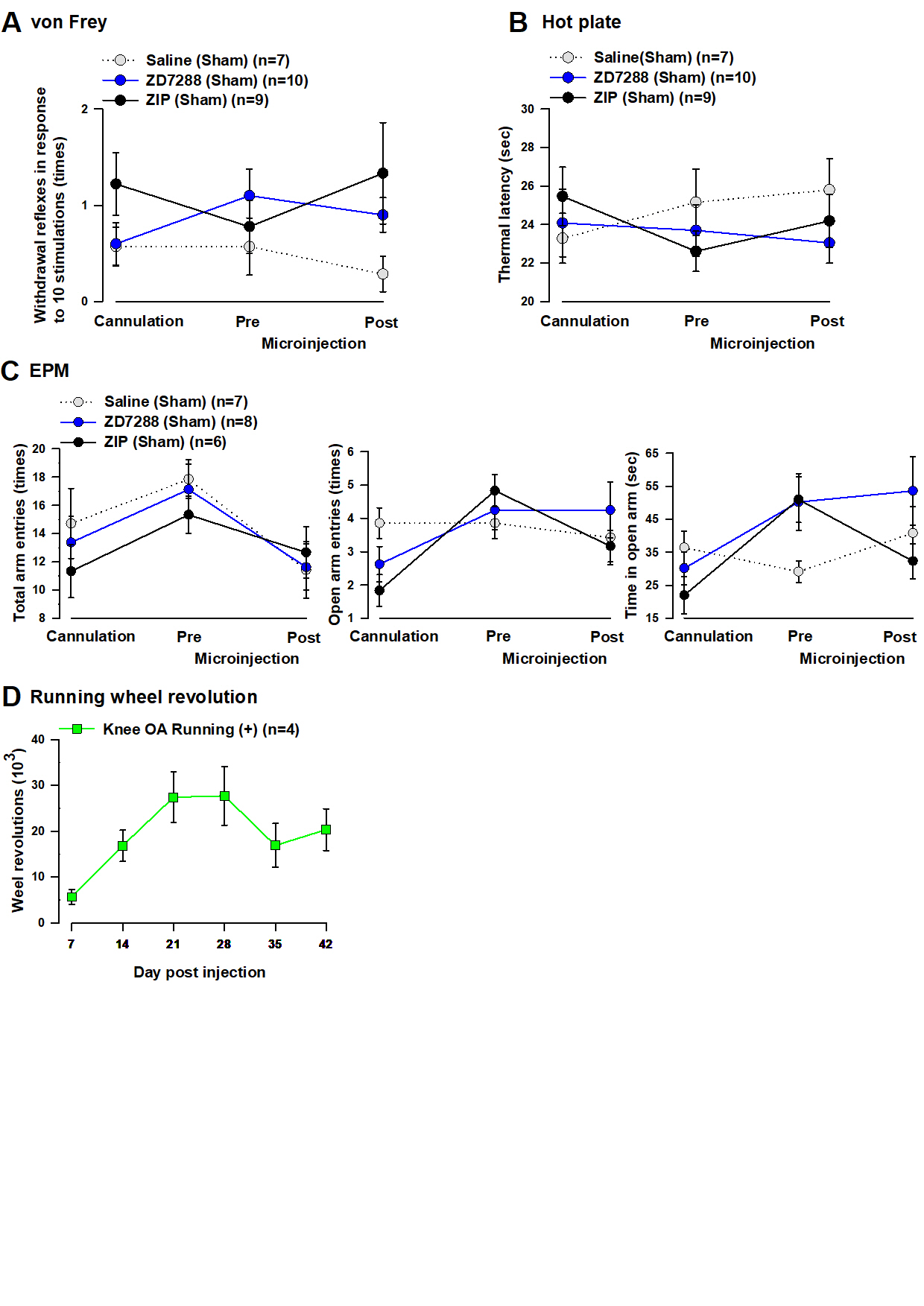

Supplement: Supplementary file 1 — Additional file 1. Sham mice did not exhibit pain-escape or anxiety-like behaviors, which did not change after microinjection of ZD7288 and ZIP into the ACC. (A) After microinjection, the sham (ZIP microinjection) group (black circle: n = 9 mice) showed no significant difference in the number of withdrawal reflexes of the right hindlimb compared to the sham (saline microinjection) group (gray circle: n = 7 mice). In addition, after microinjection, the sham (ZD7288 microinjection) group (blue circle: n = 10 mice) showed no significant difference in the number of withdrawal reflexes of the right hindlimb compared to the sham (saline microinjection) group (gray circle: n = 7 mice). (B) After microinjection, the sham (ZIP microinjection) group (black circle: n = 9 mice) showed no significant difference in the time until the appearance of a noxious sign compared to the sham (saline microinjection) group (gray circle: n = 7 mice). Moreover, after microinjection, the sham (ZD7288 microinjection) group (blue circle: n = 10 mice) showed no significant difference in the time until the appearance of a noxious sign compared to the sham (saline microinjection) group (gray circle: n = 7 mice). (C) After microinjection, the sham (ZD7288 microinjection) group (blue circle: n = 8 mice) showed no significant difference in the number of total arm entries, open arm entries, and the time staying in the arm compared to the sham (saline microinjection) group (gray circle: n = 7 mice). (D) The number of running wheel revolutions in the knee OA (+ voluntary running exercise load) group (green square: n = 4 mice) was 20.3 (103) ± 4.5 (103) revolutions from 5–6 weeks after voluntary running exercise load. [file 13041_2025_1207_MOESM1_ESM.docx]
